# Prevalence and Associated Factors of *optrA*-Positive-*Enterococcus faecalis* in Different Reservoirs around Farms in Vietnam

**DOI:** 10.3390/antibiotics12060954

**Published:** 2023-05-24

**Authors:** Hoang Thi An Ha, Phuong Thi Lan Nguyen, Tran Thi Mai Hung, Le Anh Tuan, Bui Thanh Thuy, Tran Hoang My Lien, Pham Duy Thai, Nguyen Ha Thanh, Vu Thi Ngoc Bich, Tran Hai Anh, Ngo Thi Hong Hanh, Nguyen Thi Minh, Duy Pham Thanh, Si-Nguyen T. Mai, Hao Chung The, Nguyen Vu Trung, Nguyen Hoai Thu, Tran Nhu Duong, Dang Duc Anh, Pham Thi Ngoc, Anne-Laure Bañuls, Marc Choisy, H. Rogier van Doorn, Masato Suzuki, Tran Huy Hoang

**Affiliations:** 1 Hanoi Medical University, Hanoi 100000, Vietnam; anha@vmu.edu.vn (H.T.A.H.); haianhtran.med@gmail.com (T.H.A.); 2 Department of Microbiology, Vinh Medical University, Vinh 431000, Vietnam; 3 National Institute of Hygiene and Epidemiology, Hanoi 100000, Vietnam; ntlp@nihe.org.vn (P.T.L.N.); ttmh@nihe.org.vn (T.T.M.H.); lat@nihe.org.vn (L.A.T.); thuybui099@gmail.com (B.T.T.); mymylien272@gmail.com (T.H.M.L.); phamduythai8289@gmail.com (P.D.T.); hathanh160199@gmail.com (N.H.T.); ngohanh_85@yahoo.com (N.T.H.H.); ntm@nihe.org.vn (N.T.M.); tnd@nihe.org.vn (T.N.D.); dda@nihe.org.vn (D.D.A.); 4 Oxford University Clinical Research Unit, Hanoi 100000, Vietnam; bichvtn@oucru.org (V.T.N.B.); rvandoorn@oucru.org (H.R.v.D.); 5 Oxford University Clinical Research Unit, Ho Chi Minh City 700000, Vietnam; duypt@oucru.org (D.P.T.); nguyenmts@oucru.org (S.-N.T.M.); haoct@oucru.org (H.C.T.);; 6 Pasteur Institute in Ho Chi Minh City, Ho Chi Minh City 700000, Vietnam; trungnv@pasteurhcm.edu.vn; 7 PATH, Hanoi 100000, Vietnam; thnguyen@path.org; 8 National Institute of Veterinary Research, Hanoi 100000, Vietnam; minhgoc27169@gmail.com; 9 MIVEGEC (IRD-CNRS-Université de Montpellier), LMI DRISA, Centre IRD, 34394 Montpellier, France; anne-laure.banuls@ird.fr; 10 Centre for Tropical Medicine and Global Health, Nuffield Department of Clinical Medicine, University of Oxford, Oxford OX1 4BH, UK; 11 National Institute of Infectious Diseases, Tokyo 162-0052, Japan; suzuki-m@niid.go.jp

**Keywords:** *E. faecalis*, farms, flies, linezolid resistance, *optrA* livestock, Vietnam

## Abstract

Linezolid is an antibiotic of last resort for the treatment of infections caused by Gram-positive bacteria, including vancomycin-resistant enterococci. *Enterococcus faecalis*, a member of enterococci, is a significant pathogen in nosocomial infections. *E. faecalis* resistance to linezolid is frequently related to the presence of *optrA,* which is often co-carried with *fex*, phenicol exporter genes, and *erm* genes encoding macrolide resistance. Therefore, the common use of antibiotics in veterinary might promote the occurrence of *optrA* in livestock settings. This is a cross-sectional study aiming to investigate the prevalence of *optrA* positive *E. faecalis* (OPEfs) in 6 reservoirs in farms in Ha Nam province, Vietnam, and its associated factors and to explore genetic relationships of OPEfs isolates. Among 639 collected samples, the prevalence of OPEfs was highest in flies, 46.8% (51/109), followed by chickens 37.3% (72/193), dogs 33.3% (17/51), humans 18.7% (26/139), wastewater 16.4% (11/67) and pigs 11.3%, (14/80). The total feeding area and total livestock unit of the farm were associated with the presence of OPEfs in chickens, flies, and wastewater. Among 186 OPEfs strains, 86% were resistant to linezolid. The presence of *optrA* was also related to the resistant phenotype against linezolid and levofloxacin of *E. faecalis* isolates. Close genotypic relationships identified by Pulsed Field Gel Electrophoresis between OPEfs isolates recovered from flies and other reservoirs including chickens, pigs, dogs, and wastewater suggested the role of flies in the transmission of antibiotic-resistant pathogens. These results provided warnings of linezolid resistance although it is not used in livestock.

## 1. Introduction

Linezolid is the first synthetic antimicrobial agent of the oxazolidinone class. It inhibits primary ribosome synthesis and protein synthesis of many species of Gram-positive bacteria by targeting the 50S ribosomal subunits and acting on its binding affinity to N-formylmethionyl-tRNA [1]. Because of its broad antibacterial spectrum, linezolid has been widely used as one of the main options for the treatment of infectious diseases caused by multidrug-resistant Gram-positive pathogens, including methicillin-resistant *Staphylococcus aureus* (MRSA), vancomycin-resistant enterococci (VRE), penicillin-resistant *Streptococcus*, and *Mycobacteria* [2]. However, there is an alarming increase in resistance to linezolid, especially in livestock farming. In Korea, during the period of 2010–2019, the rate of linezolid-resistant *E. faecalis* escalated from 0% to 5.7% in pigs and from 0% to 2.2% in chicken [3].

Although members of the *Enterococcus* genus are considered commensal bacteria, they can also be opportunistic pathogens in favorable environmental conditions. Hospital-acquired intestinal infections have become a cause of global concern due to their increasing morbidity and resistance to several important classes of antibiotics, such as vancomycin, linezolid, and fluoroquinolone. Notably, these resistances in many cases came from farm animals [4,5]. *E. faecalis* is ubiquitous bacteria in the intestinal tract of mammals and it is also a significant pathogen in foodborne infections and antibiotic resistance. With many virulent factors that help to adhere, penetrate, and evade the immune response, the pathogenic potential of *E. faecalis* is higher than other *Enterococcus* species. It can be responsible for endocarditis, sepsis, wound infections, and urinary tract infections [6,7,8,9,10]. Due to its intrinsic resistance to antibiotics such as cephalosporins, sulfonamides, and clindamycin and acquisition of other resistance genes through mobile genetic elements (plasmids, transposons) such as *vanA*, *vanB* (vancomycin resistance), *ermA, ermB* (macrolide resistance), *fexA* (phenicol resistance) or by mutations under the selective pressure of antibiotics (daptomycin, fluoroquinolone resistance), its resistance is even more worrying [11,12]. Multiple-drug resistant (MDR) *E. faecalis* is reported worldwide and presents an increasing trend in both hospitals and communities, in animal food and farming settings [3,8,13,14]. In Vietnam, Indonesia, and Thailand (2007), 73–100% of *E. faecalis* isolates obtained from chickens exhibited MDR [15].

Linezolid resistance can be caused by (i) mutations in the V domain of 23S rRNA and *rplC/rplD* genes encoding the L3/L4 ribosomal proteins, (ii) acquisition of oxazolidinone resistance genes such as *cfr*, which encodes a methyltransferase-modified 23S rRNA, or (iii) acquisition of *optrA* and *poxtA*, two genes encoding the ABC-F protein that presumably protects the ribosomal target from binding to antibiotics [16]. Among genes encoding resistance to linezolid reported in *E. faecalis*, *optrA* is the most common and was reported worldwide [17,18,19]. This gene was detected in both plasmids, chromosomes, and different genetic environments [14,18,19]. Multiple variants of the gene have been described, such as *optrA2*, *optrA5*, *optrA7*, etc., demonstrating the plasticity of this resistance region [20,21]. *OptrA* confers transferable resistance to linezolid and is often co-carried with *fexA*, *fexB* (phenicol exporter genes), and *ermA*, *ermB* (conferring macrolide-licosamide-streptogramin B resistance) [17,21,22,23]. Therefore, although linezolid is not permitted in livestock, common use of other antibiotics in veterinary might promote the occurrence of *optrA* in animal reservoirs [24,25,26]. Furthermore, *OptrA* regularly locates on plasmids with a capacity to transmit horizontally between bacteria, creating a greater risk of getting resistance genes [22,27].

The use of antibiotics in livestock increases selection pressure on bacteria and generates the development of antibiotic resistance in animals. In addition, poor living conditions (improper lavatories, untreated wastewater, poor hygiene, etc.) and close interactions between farmers with food animals and the farm environment can facilitate the transmission of resistant bacteria, including *Enterococcus*, from animals to humans and vice versa [28,29,30,31]. The emergence of antimicrobial resistance in the livestock industry, especially resistance to critically important antimicrobials such as linezolid [32], poses a serious threat to public health [33]. Linezolid-resistant *E. faecalis* and *optrA*-positive *E. faecalis* (OPEfs) have been detected in many countries in livestock and animal food [18,27,34] but data on associations between OPEfs infection in the community and its associated factors are scarce. Only some reports on the correlation between linezolid resistance and clinical epidemiological factors were published [8,9,35].

In Vietnam, linezolid is not approved for veterinary use in livestock [36]. The data regarding linezolid resistance in communities and farms are, thus, relatively scarce and mostly from clinical settings. Furthermore, studies on antibiotic resistance in healthy humans or animals typically concentrate on Gram-negative pathogens such as *E. coli* or *Salmonella* [37,38] while only a few studies focus on enterococci. Data on linezolid-resistant *E. faecalis* and *optrA* gene are even more limited. Therefore, it is essential to understand the presence and transmission of *optrA-positive E. faecalis* among humans and reservoirs connected to farm animals. This study aims to (1) investigate the prevalence of *optrA-positive E. faecalis* (OPEfs) in humans, chickens, pigs, dogs, wastewater, and flies on farms and (2) identify factors associated with the presence of this pathogen and genetic relationships of OPEfs isolates in livestock settings.

## 2. Results

### 2.1. Demographic Characteristics

Out of 139 enrolled farmers 78 were male (56.1%). Most of them were over 40 years old (82%), in secondary school in literacy (64.7%). More than two-thirds of farmers (70.5%) worked at household farms, 17.3% on small farms, and 12.2% on medium farms. There was no large farm in our study. Among 70 investigated farms, 72.9% were households, 17.1% were small and 10% were medium farms. The total livestock unit of farms ranges from 0.2 to 37.0 with a mean of 9.6. Poultry farms took 65.7% (46 farms), only 4 pig farms and 20 mixed farms (Table 1).

### 2.2. Prevalence of E. faecalis and OPEfs in Collected Samples

**Figure 1:**
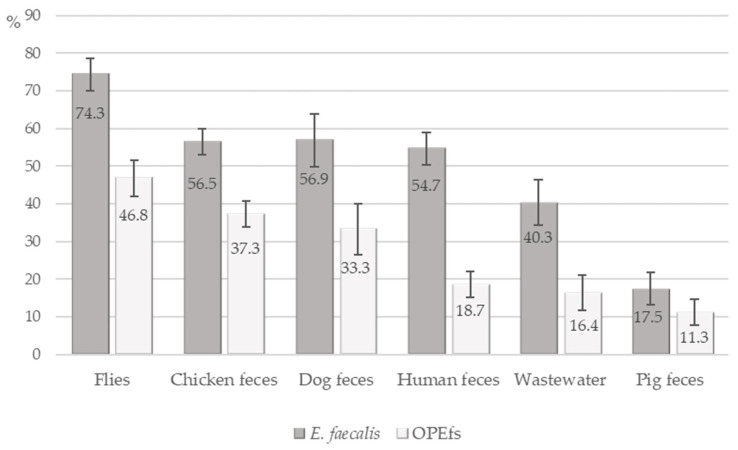
Detection of *E. faecalis* and *OptrA*-positive *E. faecalis* in samples.

A total of 639 samples from investigated farms were collected, including feces samples from humans (n = 139, from 70 farms), chickens (n = 193, from 66 farms), pigs (n = 80 from 24 farms), and dogs (*n* = 51 from 51 farms); wastewater and flies (*n* = 67 and 109, from 67 farms). Out of 639 tested samples, 336 (52.6%) samples contained *E. faecalis* and 186 (29.1%) samples contained *optrA*-positive *E. faecalis* (OPEfs). Figure 1 shows the distribution of *E. faecalis* and OPEfs in 6 sample types. The highest occurrence of *E. faecalis* and OPEfs was observed in flies at 74.3% (81/109) and 46.8% (51/109), respectively), compared to chickens (56.5% (109/193) and 37.3% (72/193)), dogs (56.9% (29/51) and 33.3% (17/51)), and in wastewater (40.3% (27/67) and 16.4% (11/67)). These proportions were lowest in pigs (17.5% (14/80) and 11.3% (9/80)). The prevalences in humans were 54.7% (76/139) and 18.7% (26/139), respectively. By age, 21.5% of farmers in the 41–60 age group (17/62) harbored OPEfs, higher than other groups. By education level, farmers with high school and above had the lowest OPEfs infection rate (11.4%, 3/41) (Table 2).

**Figure 2:**
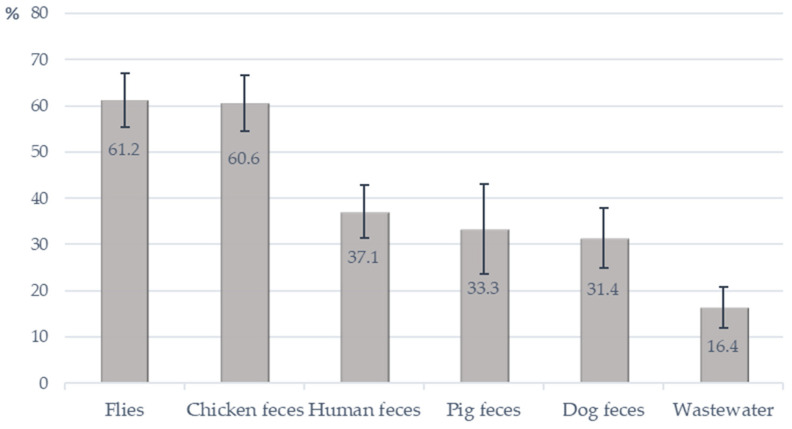
Detection of *OptrA*-positive *E. faecalis* from investigated farms.

At the farm level, the prevalence of OPEfs was also highest in fly samples with 41 of 67 investigated farms having OPEfs positive samples (61.2%). The lowest prevalence was in wastewater (16.4%, 11/67 farms). Twenty-six of 70 farms (37.1%) had farmers harboring OPEfs (Figure 2).

### 2.3. Associated Epidemiology Factors of OPEfs in Different Sample Types

Epidemiological factors were analyzed for 6 investigated objects (Table 2). In humans, there was no statistically significant association between the investigated epidemiological factors and OPEfs carriage except the farm scale factor. Farmers working on small farms were 2.77 times more likely to be infected with OPEfs than those working on household farms (OR = 2.77, *p* = 0.049).

The total livestock unit of the farm did not affect OPEfs status in humans but was associated with carriage status in chickens, flies, and wastewater. An increase of the total livestock unit of a farm by 1 unit was likely to increase the possibility of OPEfs carriage in these objects by 3% (*p* = 0.02), 7% (*p* = 0.002), and 8% (*p* = 0.005), respectively. Univariate analysis showed that feeding area was a factor frequently associated with OPEfs infection in 3 reservoirs, excluding human, dog, and pig fecal samples. Particularly, chickens on farms with over 1000 square meters had a nearly 2 times greater risk of getting OPEfs than those on smaller farms (OR = 1.94, *p* = 0.04). The risks were even higher for flies (OR = 2.85, *p* = 0.02) and wastewater samples (OR = 4.4, *p* = 0.03). However, the multivariate logistic regression model rejected the association of these factors with infection status in chickens and wastewater (*p* > 0.5). In flies, total livestock unit of farm and antibiotic use in animals were association factors for OPEfs. When the total livestock unit of farm increased by one unit, flies were 6% more likely to be infected with OPEfs (*p* = 0.04) and flies in farms where farmers used antibiotics in livestock brought OPEfs more than 3.35 times than farms that did not use (*p* = 0.02). There was no statistically significant relationship between investigated factors and OPEfs infection in pigs and dogs. Therefore, data from these samples are not shown.

### 2.4. OptrA-Positive E. faecalis Status and Antibiotic Resistance

The antibiotic susceptibility of 336 *E. faecalis* strains obtained from all samples was tested by using the agar dilution method. There was no strain resistant to penicillin, ampicillin, and vancomycin. However, 53 strains (15.8%) showed an intermediate level of sensitivity to vancomycin. Antibiotic resistance rates to the remaining 10 antibiotics were presented in Table 3. The highest resistance rate was observed for tetracyclin (85.7%), followed by doxcycyclin (76.8%) and erythromycin (61.9%). The highest resistance rate specified by sample types also belonged to tetracycline. Resistance rates were higher in the *optrA*-positive group for all tested antibiotics and all types of samples.

Overall resistance and intermediate rate to linezolid in all *E. faecalis* strains were 48.8% (164/336) and 3.6% (12/336), respectively. The resistance rate to linezolid was highest in pigs (64.3%), followed by chickens (60.6%) and flies (54.3%). In humans, linezolid-resistant *E. faecalis* was 31.6% (24/76) of obtained strains. Among 186 OPEfs strains, 86% (160) isolates were resistant to linezolid while only 2.7% (4/150) *optrA*-negative-*E. faecalis* strains had resistance phenotype.

Multidrug resistance (MDR) *E. faecalis* was detected as shown in Table S1. The majority of OPEfs isolates (86.0%, 160/186 strains) had an MDR pattern. 82.3% of these isolates were resistant to 5 and above tested antibiotics, and 19 isolates were resistant to 9 over 13 tested antibiotics from different classes including tetracycline, macrolide, phenicol, fluoroquinolone, and linezolid. Two OPEfs isolates, recovered from flies, were found to be resistant to 10 antibiotics. The rate of MDR in OPEfs was significantly higher than in the *optrA*-negative *E. faecalis* group. Only 37 out of 150 (24.7%) *optrA*-negative strains were MDR, with 1 strain being resistant to 8 antibiotics. 19.3% of this group were not resistant to any antibiotics, while only 1.1% of OPEfs strains had this phenotype. Generally, the MDR rate in all *E. faecalis* isolates was 58.6% (197/336).

Presence of *optrA* on *E. feacalis* was significantly associated with resistant phenotype to linezolid (OR = 448) and also to other antibiotics (OR ranged from 2.15 for ciprofloxacin to 9.07 for high-level streptomycin resistance), except for vancomycin. Multivariate analysis confirmed positive associations between the presence of *optrA* on *E. feacalis* and their resistant phenotype to linezolid (adjusted OR = 540) and levofloxacin (adjusted OR = 5.37) (Table 4).

### 2.5. Molecular Typing of optrA-Positive E. faecalis by PFGE

**Figure 3:**
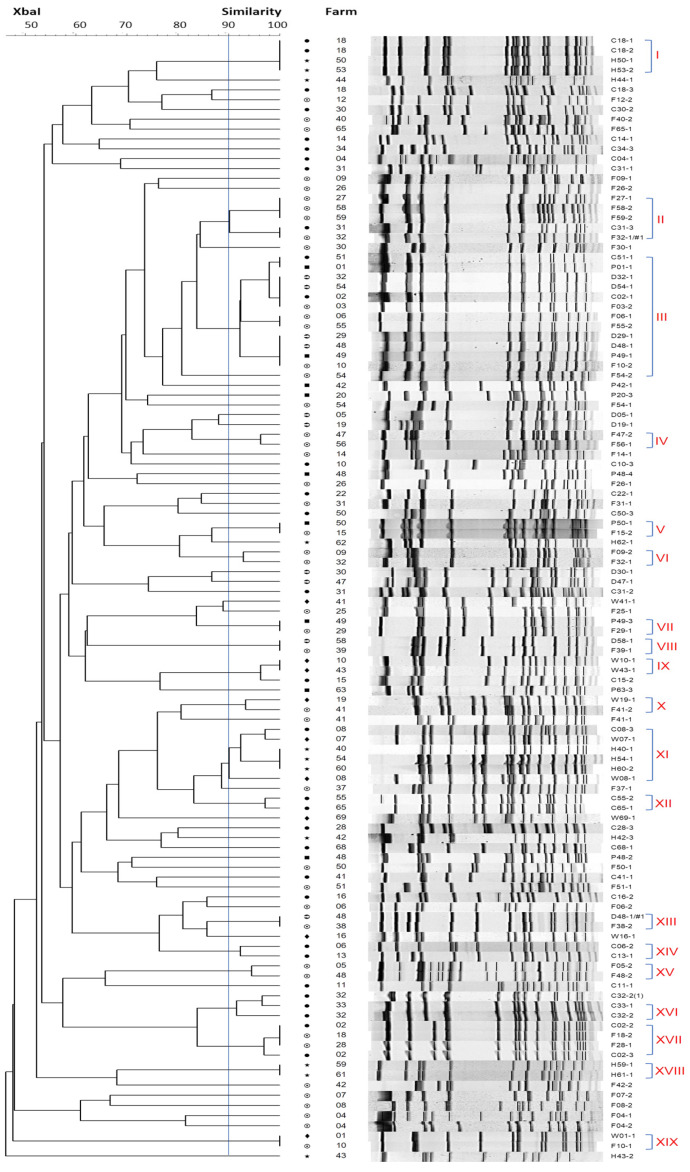
Genetic relatedness of the 114 *optrA*-positive *E. faecalis* examined, based on the PFGE banding patterns of the isolates. Strain codes and PFGE subtypes are depicted. A total of 19 pulsotypes were identified at 90% similarity, designated as I through XIX. (C: chickens, D: dogs, F: flies, H: humans, P: pigs, W: wastewater).

114 OPEfs isolates obtained from 59 farms were chosen for PFGE. Genetic relationships among them were illustrated in Figure 3. PFGE analysis with a similar cut-off value at 90% revealed 72 types of pulse patterns with a low degree of homology between lines. 19 clusters were shared pulse types (designated as I through XIX) and 53 were treated as unique. A total of 34 pulsotypes for 44 isolates from flies, 7 for 11 isolates from humans, and 8 for 9 isolates from wastewater were obtained. In animal fecal samples, 31 isolates from chickens, 10 from dogs, and 9 from pigs were divided into 24, 7, and 8 pulsotypes respectively. There were differences in PFGE patterns between isolates from flies and humans but had overlapping PFGE types between flies and pigs (clusters III, V, VII), flies and dogs (clusters III, VIII, XIII), flies and chickens (clusters II, III, XVII), and flies and wastewater (clusters X, XIX). In cluster II, 3 isolates of flies had a similar type of pulse to 2 isolates from chickens. There were up to 12 OPEfs strains from dogs (4 strains), chickens (2 strains), pigs (2 strains), and flies (4 strains) obtained from 12 farms that had similar pulse patterns after electrophoresis in cluster III. Only types IV, VI, XII, XIV, XV and XVI had shared pulse types between the same sample types (2 samples per cluster). Isolates from the same farm did not cluster together with the exception of 4 isolates from chickens in cluster I (farm 18) and XVII (farm 2). In humans, there were no share PFGE patterns of OPEfs strains discovered from dogs, pigs, and flies but had a close genetic relationship between 4 isolates collected from farmers on farms 50, 53 and chicken on farm 18 (cluster I), 6 isolates from farms 50, 54, 60 (humans), farm 7,8 (wastewater) and farm 8 (chickens) (cluster XI). Farmers in farms 59 and 61 harbored OPEfs strains with identical pulsotype.

## 3. Discussion

This is one among a few studies in Vietnam utilizing the One Health approach in investigating the situation and transmission of antibiotic-resistant bacteria in livestock settings. One Health concept was first mentioned in 2003–2004 in accordance with the emergence of SARS and avian influenza H5N1 [39]. One Health approach recognizes the interconnection between humans with shared environments with animals and plants. This approach is getting more critical in recent years and its health issues include emerging and re-emerging zoonotic diseases, vector-borne diseases as well as other health threats to humans, animals, and the environment [40]. Antibiotic resistance is also an issue of One Health. The global action plan on antimicrobial resistance in 2015 confirms that antibiotic resistance affects all areas of health and has impacts on various sectors and on the whole of society. The plan also underscores the roles of various sectors and resources to combat antibiotic resistance [41].

First identified in China in *E. faecalis* and *E. faecium* isolates in 2015 [17], resistance to oxazolidinones mediated by *optrA* is now detected worldwide, from both clinical resources, healthy humans, and animals [14,18,42]. However, in Vietnam, intensive research on the *optrA* gene and linezolid-resistant *E. faecalis* in clinical and community as well as associated factors are limited. According to our best knowledge, this is the first report that demonstrates the prevalence of OPEfs in livestock in our country. *E. faecalis* and OPEfs were present in all types of samples, from farmers, and animals (dogs, chickens, pigs) to flies and wastewater. It showed a higher prevalence of *optrA* in isolates from animals rather than humans. According to other studies [21,43,44], whole genome sequencing results indicated that *fexA* was co-located with *optrA* in *E. faecalis* strains, suggesting that *optrA*, a gene associated with resistance to linezolid, may be selected due to non-oxazolidinone antibiotics usage, such as phenicol (thiamphenicol, florfenicol, etc.). The popular use of phenicol in prevention and treatment of diseases in animals in Vietnam may explain the high percentage of OPEfs in this study [24,25,26].

In humans, the study indicated 18.7% of healthy farmers were infected with OPEfs, which is higher than 2.31% (36/1558) for adults and 3.47% (66/1900) for children in Hangzhou, China (2015) [44]. Study on fecal samples of humans and animals collected from 1998–2014 in five provinces/cities (Shandong, Henan, Tibet, Guangdong, and Shanghai), Wang reported a low proportion of *optrA*. Only 1.68% (10/595) of humans and 14.1% (41/290) animals (pigs n = 33; chickens n = 8) had OPEfs [17]. Compared to *E. faecalis* isolated from clinical infections in Spain in 2016–2017, and Korea in 2020, our results were higher [45,46]. In animal fecal samples, OPEfs prevalence in this study seemed more emergency than what was observed in cattle and pigs in Portugal in 2017 (0/201 and 6/249 samples) [34]. Notably, the results identified flies as the biggest host of this pathogen with 46.8% of samples infected by OPEfs. This prevalence was higher than the prevalence reported in a study on the carriage of mcr-1-positive *Escherichia coli* in Vietnam (36.7%) [29]. Carriage of resistant pathogens in flies was also reported in China [47]. The presence of OPEfs as well as other pathogens in flies might be due to exposure to various contaminated reservoirs and this implied contribution of flies to the spread of OPEfs by their borderless mobility.

No association between demographic factors such as age, sex, and education level and OPEfs infection in humans was identified in this study. Xiaoyu Ma’s study also reported no association of age with the carriage of linezolid-resistant *E. faecalis* isolated from patients with urinary tract infections in China from 2010 to 2015 [9]. However other studies reported an association of age with antibiotic-resistant bacteria infection status in humans. Jiaqi Zou studied 1902 samples that were collected in the Hospital of Chongqing Medical University for 5 years (2014–2018) and reported that patients with linezolid-resistant *Enterococcus* infections tended to be older than the control group [8]. Similarly, the older age group was also identified as at higher risk of other pathogens, such as mcr-1-positive *Escherichia coli* in a study in Vietnam [29]. The unique factor associated with OPEfs in humans was the farm scale (OR = 2.77, *p* = 0.049). The association of farm scale to infection status in farmers was also previously reported on antibiotic-resistant *E. coli* in Vietnam and Thailand [29,48].

The total livestock unit of the farm was also associated with the OPEfs infection in various reservoirs, including chicken, flies, and wastewater. Following that, an increase of the total livestock unit of the farm by 1 unit added a 3% chance of acquiring OPEfs in chicken, 7% in files, and 8% in wastewater. Similarly, the feeding area was also correlated with OPEfs status in these reservoirs. Chicken raised in over 1000 square meters of farms was 2.46 times more likely to get an infection than those in smaller farms. This ratio in flies was 2.85 and in wastewater was 4.40. It can be explained by the fact that the larger the animal feed, the more antibiotic may be used. The study in Thailand reported that medium-scale farms (having 100–500 sows) used a greater diversity of antimicrobials than small-scale farms (with a maximum of 20 sows) and also administered antimicrobials to a higher extent [48]. Similarly, two studies in Ghana identified antibiotic use in 100% and 97% of commercial farms while the percentage in backyard farms and domestic farms was only one-quarter and nearly one-half, respectively. Higher demand for productivity and a large number of animal feed in commercial farms can explain a higher use of antibiotics to maintain the wellness of animals [49,50].

Interestingly, antibiotic use in livestock did not create a statistically significant difference with OPEfs carrier status in humans and livestock but was associated with OPEfs carriage in flies. Multivariate analysis confirmed that flies captured in farms using antibiotics in livestock had a 3.35 times higher prevalence of OPEfs than those in farms that did not (*p* = 0.02). Exposure to a wide variety of samples in the environment, such as antibiotic-resistant bacteria in animal feces, wastewater, and food containing antibiotics might create this difference. Flies carrying bacteria that resist important antibiotics pose a potential threat to human health. A previous study in Vietnam also reported the potential roles of flies in the transmission of resistant pathogens in farming settings where associations of infection status with mcr-1-positive *Escherichia coli* in flies and in food animals in farms were identified [29]. They not only contact with animals, and the environment but also get into human food and water. Flies have been shown to be important vectors of the spread of antibiotic-resistant bacteria [51] and they are considered the most important non-biting insect pests in the medical and veterinary field because of the huge number of pathogens carriage [52]. Our PFGE data has confirmed this point. PFGE results demonstrated a genotypic diversity and wide transmission among OPEfs strains collected from a variety of samples in different farms (Figure 3). Among 114 OPEfs, 19 of 72 PFGE types were identified in at least 2 isolates or more. More than half of them (12/19 pulsotypes) contained OPEfs isolates from flies, which had the same PFGE subtype as others on distant farms. Flies in farms 27, 58, 59 (cluster II), farms 6, 55 (cluster III), farms 47, 56 (cluster IV), farms 09, 32 (cluster VI), and farms 05, 48 (cluster XV) provided OPEfs isolates with similar pulse types. There was no genetic relationship between OPEfs from flies and humans, but overlapping genotype between flies and dogs (2 pairs, farms (38, 48), farms (39, 58)), flies and pigs (2 pairs, farms (15, 50), farms (29, 49)), flies and chicken (2 pairs, farm (2, 18); (31, 32)), flies and wastewater (1 pair, farms 1, 10) were observed. According to Tenover et al., each pair of OPEfs strains had indistinguishable band patterns that could be “considered to represent the same strain” [53]. Especially, in cluster III, 12 isolates were collected from flies, dogs, pigs, and chickens in farms 1, 2, 3, 6, 10, 32, 48, 49, 51, 54, and 55 displayed genetic correlations at 96% to 100%. These farms were approximately 0.3 to 5.4 km distance from each other, suggesting the wide spread of resistant strains in the environment most likely through the mobility of files. Numerous studies also have demonstrated that flies can acquire, harbor, and transmit other resistant pathogens [29,54,55]. The high proportion of *E. faecalis* with *optrA* in flies in our study has strengthened the potential threat of flies to public health. Due to their unrestricted movement, and their attraction to residential areas, flies can play an important role in the ecology and transmission of bacteria, including enterococci with antibiotic-resistance genes [51,56].

In the remaining 53 unique pulse types, *E. faecalis* strains carrying the *optrA* gene were genetically heterogeneous indicating that a vast majority of them were not derived from a single clone, such as G. Dicuonzo’s study in Italy (reported in 2001) and Tamang MD’s study in Korea (published 2017) [13,27]. Exposure to physical and chemical stresses may have led to the evolution of a wide range of traits, which are necessary for the adaptation of *E. faecalis* to different environments. The evolutionary process, such as mutation, selection, and recombination might have played a role in the evolution of environmental stress tolerance, resulting in the observed high diversity [57].

Determination of sensitivity to linezolid of OPEfs strains showed resistant or intermediate ratio up to 92.5% (172 over 186 strains), proving the critical role of *optrA* to phenotypic resistance to linezolid. Univariate binary logistic regression analysis identified that *E. feacalis* strains carrying *optrA* were 448 times more likely to resist or reduce sensitivity to linezolid, multivariate analysis even strengthened this association (adjusted OR = 540, 95%CI 134-2175). Only 4 *optrA* negative *E. faecalis* resisted linezolid, which can be explained by other resistant mechanisms in these strains. Our results showed a contrast to the reported clinical data of a previous study in patients and pigs from 2008–2010 in Ha Noi (Vietnam) which reported no linezolid-resistant *E. faecalis* strain [5]. This raised an alarm about the presence and spread of resistant strains in the community. Compared to clinical data, the rate of linezolid resistance in this study is truly concerning [8,9,10].

Susceptibility testing results identified that OPEfs was not only resistant to linezolid but also to many other antibiotics commonly used in clinical settings. For vancomycin, a critical antibiotic in the treatment of positive gram bacteria infections, although there was no resistant strain identified, 15.8% of strains showed intermediate resistance to this antibiotic. Antibiotic sensitivity testing also raised an alarm of MDR *E. faecalis*, especially OPEfs strains, that showed resistance to important antibiotics such as fluoroquinolones. High-level resistance of aminoglycosides and macrolides of these strains was also reported (Table S1). Compared to a study by M. Usui et al. in 2015 on poultry farms in Southeast Asian countries, MDR rates in *E. faecalis* in our study were lower. The study pointed out that 100% of *E. faecalis* isolates in Vietnam were MDRO and was found to be resistant to 2–8 different antimicrobials, while 49 out of 58 isolates (84.5%) recovered from Indonesia exhibited MDR and resisted 2–6 antibacterial agents; 27 out of 37 isolates (73.0%) obtained from Thailand resisted to 2–7 different antibiotics [15]. However, in that study, resistance to linezolid and fluoroquinolone was not detected, and a higher MRD *E. faecalis* rate could be explained by a difference in criteria used to define multidrug-resistant bacteria. Moreover, it was noteworthy that resistance prevalence was high in flies, pigs, and chickens. As discussed above, flies are an important vector in the spread of antibiotic-resistant bacteria while chickens and pigs are 2 common sources of food in the Vietnamese diet. Therefore, MDR *E. faecalis* and OPEfs can be transmitted from animals to humans through flies or throughout the food chain. A similar MDR rate of *E. faecalis* (52.5%) was also detected in South Korea from cattle, chickens, and pigs during the period of 2010–2019 [3]. Although all OPEfs strains were susceptible to first-line treatment (ampicillin, penicillin), they were still threats to public health as reservoirs for many antibiotic-resistant genes, including *optrA*, which may horizontally spread through plasmids or transposons to other *Enterococcus*. Vice versa, OPEfs can receive other genes, for example, *bla-*, which makes resistance more worrisome.

Analyzing the relationship between *optrA* carriage status in *E. faecalis* isolates and their resistant phenotype to other antibiotics, we also found significant associations. The presence of the *optrA* gene was associated with phenotypic resistance of *E. faecalis* isolates against almost all tested antibiotics, excluding vancomycin. Multivariate analysis confirmed positive associations between the presence of *optrA* on *E. feacalis* and their resistant phenotype to levofloxacin (adjusted OR = 5.37) but rejected associations with phenotypic resistance to remaining antibiotics (Table 4). Levofloxacin is not on the list of livestock drugs allowed for trade in Vietnam [36] but there were 25% (84/336) of *E. faecalis* strains obtained in the study resisted or reduced sensitivity to this antibiotic, and there was an association between the presence of *optrA* to resistant phenotype to this antibiotic. These 2 points suggested the need to research resistance to levofloxacin in livestock settings.

The small sample size was a limitation of the study. Due to limited resources, the study was conducted in a small population (a commune). This influenced the representativeness of results on OPEfs prevalence. This might also be the reason why we were unable to identify any statistically significant relationship between OPEfs carriage in humans and in other objects such as chickens or pigs, neither could we identify any associations between any of our tested independent variables and OPEfs infection status in dog feces and pig feces samples. We believe that such associations should be identified with a larger sample size.

## 4. Materials and Methods

### 4.1. Setting and Population

A cross-sectional study was conducted in 2019 on livestock farms in a commune in Ha Nam, a province of northern Vietnam. The commune was selected as one of the communes with the largest number of domesticated animals in the province and that did not participate in any previous antibiotic resistance studies. The farming areas of this commune are geographically separated from residential areas. All farms and all their residents over the age of 18 engaged in farming were invited to participate in the study. The commune Veterinary Medicine Office made a list of farms and farmers from which 70 farms with 139 farmers who provided consent were enrolled in the study. The study created no potential harm to participants’ health as no invasive practice was conducted. They were provided with complete information on the study and provided consent to participate in the study. The study also strictly followed the ethical criteria of the Declaration of Helsinki, as well as receiving full benefits.

### 4.2. Sample Collection and Laboratory Analysis

Feces samples were collected from farmers, chickens, pigs, and dogs raised in these farms. Each farmer received a sterilized container with a spoon attached to the lid, gloves, and a biohazard bag and study staff demonstrated for home collection of their stool samples. Pooled feces of animals were collected by investigators. Dog feces sample was collected from several dunghills in each farm and then pooled in 1. Two to five pooled chicken feces samples were collected from each farm depending on the number of chickens in the farm (<1000: 2 samples, from 2000 to under 3000: 3 samples, from 3000 to under 10,000: 4 samples, from 10,000 and above 5 samples.), each sample was also pooled from feces collected from several dunghills in different areas in barns. Similarly, 3 to 5 pooled pig feces samples were collected from each farm (<10 pigs: 2 samples, from 10 to under 30: 4 samples, from 30 and above 5 samples). Additional samples from flies and wastewater were also collected on the same farms. Flies were retained using a glue board, then aseptically and individually transferred into 1.5 my Eppendorf tubes which contained 1 mL LB broth medium then pulverized using disposable plastic sticks (SPL Kore). 100 mL of wastewater was collected into a sterilized plastic bottle from each farm. All samples were stored at 4 °C after collection and transferred within 14 h to the Antimicrobial Resistance Laboratory of the National Institute of Hygiene and Epidemiology (NIHE) Vietnam for testing. Laboratory technicians took a part of about 1 g of each feces samples to cryotubes. 0.22 μm filters were used to filter wastewater samples, and filters were stored in cryotubes. All feces samples flies, and filters were stored at −80 °C until further use to ensure all samples remain stable for testing.

Each sample was spread onto Enterococcus Differential Agar Base (TITG Agar Base, Himedia, Mumbai, Maharashtra, India) with TTC Solution 1% (FD057) as a supplement. For wastewater samples, after filtering, a piece of the filter was taken and cultured in 10 mL LB broth medium at 37 °C for 6–8 h. Then, 1 full loop (10 µL) of the culture was speared onto the surface of a TITG agar. After 24 h at 37 °C, selected colonies with a deep red center and a narrow white periphery were sampled to identify *E. faecalis* by the MALDI Biotyper system (Bruker Daltonik GmbH, Bremen, Germany).

*E. faecalis* DNA was extracted by the QIAamp DNA Mini Kit (Qiagen, Hilden, Germany) following the manufacturer’s protocol. Detection of the *optrA* gene was performed with 2 primers (F: AGGTGGTCAGCGAACTAA, R: ATCAACTGTTCCCATTCA) and with the following PCR condition: 5 min at 94 °C, (1 min at 94 °C, 1 min at 48 °C, 1 min at 72 °C) × 34 cycles, 7 min at 72 °C [17]. Linezolid-resistant strain, *E. faecalis* R29-1-1 was used as a positive control, which was whole genome sequenced and confirmed *optrA*-positive by the Antimicrobial Resistance Laboratory of NIHE.

Samples that did not find out *E. faecalis* or *optrA* negative *E. faecalis* were considered OPEfs negative. A farm will be considered positive for OPEfs in chickens if at least 1 among 2–5 collected chicken feces samples collected in this farm harbors OPEfs. A similar manner is applied to the 5 remaining sample types.

### 4.3. Antimicrobial Susceptibility Testing

Antibiotic susceptibility of *E. faecalis* strains was assessed by the minimum inhibitory concentration method on Muller-Hinton agar and Brain heart infusion agar (BHI, for high-level aminoglycoside resistance) [58]. The bacteria were tested against 13 antimicrobial agents which are commonly prescribed for enterococcal infections and frequently used in farms, including ampicillin, penicillin (β-lactam), vancomycin (glycopeptide), chloramphenicol (phenicol), tetracycline, minocycline, doxycycline (tetracycline), ciprofloxacin, levofloxacin (fluoroquinolone), erythromycin (macrolide), high-level resistant aminoglycoside (gentamicin, streptomycin) as well as linezolid (oxazolidinone). Isolates showing intermediate/resistant levels of susceptibility were classified as non-susceptible [59,60]. An isolate was classified as MDR when it exhibited resistance to at least 3 antibiotics classes [59]. *E. faecalis* ATCC 29212 and *E. faecalis* ATCC 51299 were used as control strains. Susceptibility tests were interpreted according to CLSI 2022 (32 edition) [61].

These data were analyzed to identify the relationship to *otrpA* carrying on *E. faecalis* isolates.

### 4.4. Epidemiological Data Collection and Analysis

Registered participants were interviewed using a structured questionnaire. Investigated variables included demographic information (age, sex, education) and characteristics of farms (e.g., type of farm, feeding area, farms scale, farm livestock unit, use of antibiotic, …). Total livestock units of farm and farm scale were specified following the guidance of Decree 13/2020/ND-CP Detail the Law on Livestock issued by the Vietnam Government. It specifies the livestock unit coefficient for each type of food animal, ranging from 0.0003 to 1. This coefficient multiplied by the number of each respective type of animal equals “livestock unit”. The total livestock units of a farm is the sum of livestock units of all food animals types raised and it is used to categorize farm scale (Household: ≤10 units, small: from 10 to less than 30 units, medium: from 30 to less than 300, large: ≥300 units) [62]. Completed interviews and laboratory results were entered using Epidata Entry v3.1 (Denmark). SPSS 20.0 software was used for data analysis. Qualitative variables were presented as frequency, percentage (%), and 95% confidence interval (95%CI), while quantitative variables were presented as minimum, maximum, mean, and standard deviation (SD). χ^2^ test, binary logistic regression (for univariate and multivariate analysis) was performed to identify the association between OPEfs carriage of various reservoirs (humans, chickens, pigs, dogs, flies, and wastewater) and independent variables. The odds ratio (OR) and 95% confidence interval (CI) were calculated to assess the strength of the association. The Hosmer-Lemeshow test was used to evaluate the goodness of fit of the logistic regression model. The associations were considered significant with a *p*-value < 0.05. Variables that have *p*-values ≥ 0.05 in the univariate analysis were not included in the multivariable analysis.

### 4.5. Pulsed-Field Gel Electrophoresis (PFGE)

Pulsed-field gel electrophoresis was used to analyze clonal relatedness among the OPEfs isolates. We selected clinically important strains which had resistant phenotype to linezolid and at least two important antimicrobial agents for Gram-positive bacteria in the clinic, including quinolone resistance, macrolide resistance, high-level aminoglycoside resistance (HLAR) or non-sensitive to vancomycin [32]. PFGE for SmaI-digested genomic DNA was performed as described by US CDC [63] with some modifications in the DNA preparation. In brief, the bacteria cells were lysed with a combination of lysozyme (20 mg/mL) and 5 μL of recombinant lysostaphin (1 mg/mL). Plugs were incubated in cell lysis buffer (6 mM Tris HCl, 1 M NaCl, 100 mM EDTA, 0.5% Brij-58, 0.2% sodium deoxycholate, 0.5% sodium lauroylsarcosine) and proteinase K (20 mg/mL) for 3 h at 54 °C with vigorous (160 RPM) agitation. Slices of DNA plugs were digested for 4 h with 25UI SmaI at 25 °C and XbaI at 37 °C. PFGE was run using CHEF-DR II system (Bio-Rad, Hercules, California, US), pulse times 3.5 s to 23.5 s for 20 h (block 1, 6 V, 120°), running temperature 14 °C. XbaI-digested Salmonella enterica serovar Braenderup H9812 was used as the size marker. The PFGE profiles were analyzed by BioNumerics version 6.6 software (Applied Maths, Kortrijk, Belgium).

Simpson’s index of diversity (D) was calculated [64] to assess the differentiation of *E. faecalis* pulsotypes by PFGE. PFGE analysis was based on the Dice similarity coefficient and unweighted pair group method using arithmetic averages (UPGMA) clustering with position tolerance and optimization coefficient of 1.0%.

## 5. Conclusions

Our study is one of a few providing data on antibiotic resistance infection in various objects in livestock in Vietnam. This data is important and in accordance with Objective 2 of the Global action plan 2015 which emphasizes gaps in knowledge on antibiotic resistance that need to be filled to guide local, national, and regional actions [41]. The results provided strong evidence of the presence of resistant genes to last-resort antibiotics for humans such as linezolid in livestock settings although these antibiotics are not used for food animals and possibility to transmit to humans of resistant genes. This suggested a need to establish surveillance programs to monitor resistance to critically important antibiotics of commensal bacteria from farm animals to prevent their transmission to humans. Results on the role of flies in the transmission of resistance isolations among long-distance farms recommend that cleaning the barn and handling and managing animal manure properly to restrict the growth of this insect is also a way to limit the spread of antibiotic-resistant bacteria, including OPEfs. The next step of our study is performing molecular research to further investigate transmission, variants, and genetic context of *optrA* as well as find out other mechanisms of linezolid resistance.

## Figures and Tables

**Table 1:** Characteristics of investigated population and farms.

Characteristics	n	%	95% CI
*Age groups (Years, N = 139)*			
18–40	25	18.0	11.5–24.5
41–60	79	56.8	48.9–64.7
>60	35	25.2	18.0–32.4
	Min = 22	Max = 85	Mean = 51.9
*Sex (N = 139)*			
Male	78	56.1	47.7–64.2
Female	61	43.9	35.8–52.3
*Education level (N = 139)*			
Primary school	14	10.1	5.8–15.1
Secondary school	90	64.7	56.9–72.7
High school and above	35	25.2	18.0–32.4
*Total livestock unit of farm*	Min = 0.22	Max = 37.04	Mean = 9.56 SD = 11.84
*Farm scales (N = 139)*			
Household	98	70.5	62.3–77.6
Small	24	17.3	11.8–24.6
Medium	17	12.2	7.7–18.9
*Farm scales (N = 70)*			
Household	51	72.9	61.0–82.2
Small	12	17.1	9.9–29.1
Medium	7	10.0	4.8–19.8
*Farm types (N = 70)*			
Chicken	46	65.7	53.6–76.1
Swine	4	5.7	2.1–14.6
Mixed	20	28.6	19.0–40.5

**Table 2:** OptrA-positive *E. faecalis* infection in samples and associated factors.

Factors	OPEfs in Sample				OR (95% CI)	*p*	Adjusted OR (95% CI)	*p*
	Negative		Positive					
	n	%	n	%				
**Human (N = 139)**								
*Age groups*								
18–40	22	88	3	12	1		-	-
41–60	62	78.5	17	21.5	2.01 (0.54–7.53)	0.30	-	-
>60	29	82.9	6	17.1	1.52 (0.34–6.75)	0.58	-	-
*Sex*								
Female	51	83.6	10	16.4	1			
Male	62	79.5	16	17.1	1.32 (0.55–3.15)	0.54	-	-
*Education level*								
Primary school	10	71.4	4	28.6	3.1 (0.65–14.73)	0.16	-	-
Secondary school	72	80	18	20	1.94 (0.61–6.19)	0.27	-	-
High school and above	31	88.6	4	11.4	1		-	-
*Farm scales*								
Household	83	84.7	15	15.3	1		-	-
Small	16	66.7	8	33.3	2.77 (1.01–7.61)	0.049	-	-
Medium	14	82.4	3	17.6	1.19 (0.30–4.63)	0.81	-	-
*Total livestock unit of farm*								
	Mean = 9.2		Mean = 11.07		0.01 (0.98–1.05)	0.47	-	-
*Farm types*								
Chicken	74	83.1	15	16.9	0.74 (0.30–1.87)	0.53	-	-
Swine	6	75.0	2	25.0	1.22 (0.21–7.11)	0.82	-	-
Mixed	33	78.6	9	21.4	1		-	-
*Average income (per month)*								
<1 million dong	25	80.6	6	19.4	1		-	-
≥1 million dong	88	81.5	20	18.5	0.95 (0.34–2.61)	0.92	-	-
*Feeding area*								
<1000 m^2^	81	83.5	16	16.5	1		-	-
≥1000 m^2^	32	76.2	10	23.8	1.58(0.65–3.85)	0.31	-	-
*Used antibiotics in livestock*								
No	34	89.5	4	10.5	1		-	-
Yes	79	78.2	22	21.8	2.37 (0.76–7.39)	0.12	-	-
*Used industrial food in livestock*								
No	12	100	0	0	-	-	-	-
Yes	101	79.5	26	20.5	-	-	-	-
**Chicken (N = 193)**								
*Farm scales*								
Household	90	66.2	46	33.8	1		-	-
Small	22	61.1	14	38.9	1.25 (0.58–2.66)	0.57	-	-
Medium	9	41.9	12	75.1	2.61 (1.02–6.64)	0.04	-	-
*Total livestock unit of farm*								
	Mean = 7.8		Mean = 11.8		1.03 (1.00–1.05)	0.02	1.01 (0.98–1.05)	0.40
*Farm types*								
Chicken	85	61.6	53	38.4	1.18 (0.62–2.20)	0.62	-	-
Mixed	36	65.5	19	34.5	1		-	-
*Average income (per month)*								
<1 million dong	26	51.0	25	49.0	1.94 (1.01–3.73)	0.04	1.40 (0.68–2.87)	0.36
≥1 million dong	95	66.9	47	33.1	1		1	
*Feeding area*								
<1000 m^2^	95	68.8	43	31.2	1		1	
≥1000 m^2^	26	47.3	29	52.7	2.46 (1.30–4.67)	0.006	1.77 (0.77–4.06)	0.18
*Used antibiotics in livestock*								
No	26	61.9	16	38.1	1		-	-
Yes	95	62.9	56	37.1	0.96 (0.47–1.94)	0.9	-	-
*Used industrial food in livestock*								
No	15	83.3	3	16.7	1		-	-
Yes	106	60.6	69	39.4	3.26 (0.91–11.6)	0.07	-	-
**Flies (N = 109)**								
*Farm scales*								
Household	50	61.7	31	38.3	1		-	-
Small	6	35.3	11	64.7	2.96 (1.00–8.80)	0.51	-	-
Medium	2	18.2	9	81.8	7.26 (1.47–35.82)	0.02	-	
*Total livestock unit of farm*								
	Mean = 4.84		Mean = 12.2		1.07 (1.03–1.12)	0.002	1.06 (1.00–1.12)	0.04
*Farm types*								
Chicken	30	45.5	36	54.5	2.22 (0.97–5.08)	0.06	-	-
Swine	4	66.7	2	33.3	0.92 (0.15–5.73)	0.93	-	-
Mixed	24	64.9	13	35.1	1		-	-
*Average income (per month)*								
<1 million dong	10	40.0	15	60.0	2.0 (0.80–4.97)	0.13	-	-
≥1 million dong	48	57.1	36	42.9	1		-	-
*Feeding area*								
<1000 m^2^	48	60.0	32	40.0	1		1	
≥1000 m^2^	10	34.5	10	65.5	2.85 (1.17–6.92)	0.02	0.82 (0.23–3.00)	0.77
*Used antibiotics in livestock*								
No	24	77.4	7	22.6	1		1	
Yes	34	43.6	44	56.4	4.44 (1.71–11.51)	0.002	3.35 (1.24–9.09)	0.02
*Used industrial food in livestock*								
No	7	63.6	4	36.4	1			-
Yes	51	52.0	47	48.0	1.61 (0.44–1.86)	0.47		-
**Wastewater (N = 67)**								
*Farm scales*								
Household	43	89.6	5	10.4	1		-	-
Small	10	83.3	2	16.7	1.72 (0.29–10.18)	0.55	-	-
Medium	3	42.9	4	57.1	11.47 (1.97–66.66)	0.007	-	-
*Total livestock unit of farm*								
	Mean = 7.14		Mean = 18.66		1.08 (1.02–1.13)	0.005	1.07 (0.99–1.13)	0.052
*Farm types*								
Chicken	35	77.8	10	22.2	4.86 (0.57–41.10)	0.15	-	-
Swine	4	100	0	0.0	0		-	-
Mixed	17	94.4	1	5.6	1		-	-
*Average income (per month)*								
<1 million dong	13	76.5	4	23.5	1.89 (0.48–7.48)	0.36	-	-
≥1 million dong	43	86.0	7	14.0	1		-	-
*Feeding area*								
<1000 m^2^	44	89.8	5	10.2	1		1	
≥1000 m^2^	12	66.7	6	33.3	4.40 (1.14–16.9)	0.03	1.7 (0.28–9.08)	0.59
*Used antibiotics in livestock*								
No	16	94.1	1	5.9	1		-	-
Yes	40	80	10	20	4.0 (0.47–33.85)	0.20	-	-
*Used industrial food in livestock*								
No	4	80	1	20	1		-	-
Yes	52	83.9	10	16.1	0.77 (0.08–7.62)	0.82	-	-

**Table 3:** Prevalence of antimicrobial resistance phenotype in *E. faecalis*.

Sample Types	*optrA*	n	Resistance to Investigating Antimicrobials																			
			TE		MN		DXT		E		C		CIP		LEV		HLGR		HLSR		LNZ	
			n	%	n	%	n	%	n	%	n	%	n	%	n	%	n	%	n	%	n	%
Human feces	Pos	26	19	73.1	15	57.7	18	63.9	12	46.2	12	46.2	1	3.8	1	3.8	2	7.7	5	19.2	22	86.4
	Neg	50	33	66.0	20	40.0	28	56.0	14	28.0	12	24.0	1	2.0	1	2.0	1	2.0	0	0.0	2	4.0
	Total	76	52	68.4	35	46.0	46	60.5	26	34.2	24	31.6	2	2.6	2	2.6	3	3.9	5	6.6	24	31.6
Chicken feces	Pos	72	70	97.2	68	62.4	65	90.3	64	88.9	66	91.7	20	28.7	16	22.2	11	15.3	31	43.1	65	90.3
	Neg	37	28	75.7	18	43.2	25	76.6	13	35.1	5	13.5	2	5.4	0	0.0	1	2.7	4	10.8	1	2.7
	Total	109	98	89.9	86	78.9	90	82.6	77	70.6	71	65.1	22	20.2	16	14.7	12	11.0	35	32.1	66	60.6
Pig feces	Pos	9	9	100	6	66.7	8	88.9	8	88.9	6	66.7	2	22.2	2	22.2	2	22.2	7	77.8	9	100
	Neg	5	5	100	3	60.0	4	80.0	4	80.0	1	20.0	1	20.0	1	20.0	0	0.0	1	20	0	0.0
	Total	14	14	100	9	64.3	12	85.7	12	85.7	7	50.0	3	21.4	3	21.4	2	14.3	8	57.1	9	64.3
Dog feces	Pos	17	13	76.5	7	41.2	11	64.7	10	58.8	10	58.8	2	11.8	2	11.8	3	17.6	7	41.2	12	70.6
	Neg	12	10	83.3	4	33.3	6	50.0	5	41.7	4	33.3	0	0.0	0	0.0	0	0.0	1	8.3	0	0.0
	Total	29	23	79.3	11	37.9	17	58.6	15	51.7	14	48.3	2	6.9	2	6.9	3	10.3	8	27.6	12	41.4
Flies	Pos	51	51	100	50	98.0	50	98.0	49	96.1	43	84.3	20	39.2	17	33.3	10	19.6	24	47.1	43	84.3
	Neg	30	26	86.7	20	66.7	23	76.7	13	43.3	6	20	1	3.3	1	3.3	0	0.0	0	0.0	1	3.3
	Total	81	77	95.1	70	86.4	73	90.1	62	76.5	49	60.5	21	25.9	18	22.2	10	12.3	24	29.6	44	54.3
Waste- water	Pos	11	10	90.9	9	81.8	10	90.9	10	90.9	10	90.9	2	18.2	1	9.1	6	54.5	8	72.2	9	81.1
	Neg	16	14	87.5	13	81.2	10	62.5	6	37.5	7	43.8	1	6.2	1	6.2	3	18.8	6	37.5	0	0.0
	Total	27	24	88.9	22	81.5	20	74.0	16	59.3	17	63.0	3	11.1	2	7.4	9	33.3	14	51.9	9	33.3
All samples	Pos	186	172	92.5	139	74.7	162	87.1	153	82.3	147	79.0	47	25.3	39	21.0	34	18.3	82	44.1	160	86.0
	Neg	150	116	77.3	76	50.7	96	64.0	55	36.7	35	23.3	6	4.0	4	2.7	5	3.3	12	8.0	4	2.7
	Total	336	288	85.7	215	64.0	258	76.8	208	61.9	182	54.2	53	15.8	43	12.8	39	11.6	94	28.0	164	48.8

(Pos: positive, Neg: negative, TE: tetracyclin, MN: minocyclin, DXT: doxycyclin, E: erythromycin, C: chloramphenicol, CIP: ciprofloxacin, LEV: levofloxacin, HLGR: high-level gentamicin resistance, HLSR: high-level streptomycin resistance, LNZ: linezolid).

**Table 4:** Relationship between harboring *optrA* in *E. faecalis* and resistance phenotype.

Antibiotic Resistance		*E. faecalis* Harbouring *optrA*				OR (95% CI)	*p*	Adjusted OR (95% CI, *p*)	*p*
		Positive		Negative					
		n	%	n	%				
Vancomycin	NS	34	64.2	19	35.8	1.5 (0.84–2.83)	0.16	-	-
	S	152	53.7	131	46.3	1		-	-
Erythromycin	NS	177	60.8	114	39.2	6.21 (2.88–13.38)	0.000	1.01 (0.19–5.45)	0.99
	S	9	20	36	80	1			
Tetracycline	NS	172	59.3	118	40.7	3.33 (1.7–6.51)	0.000	0.01 (0–0.32)	0.01
	S	14	30.4	32	69.6	1			
Doxycycline	NS	173	59.7	117	40.3	3.75 (1.89–3.73)	0.000	8.07 (0.41–157.54)	0.17
	S	13	28.3	33	71.1	1			
Minocycline	NS	170	60.1	113	39.9	3.48 (1.85–6.55)	0.000	16.99 (0.92–312.10)	0.06
	S	16	30.2	37	69.8	1			
Chloramphenicol	NS	158	68.7	72	31.3	6.11 (3.66–10.20)	0.000	2.70 (0.73–9.99)	0.14
	S	28	26.4	78	73.6	1			
Ciprofloxacin	NS	112	64.4	62	35.6	2.15 (1.39–2.33)	0.000	0.53 (0.15–1.92)	0.33
	S	74	45.7	88	54.3	1			
Levofloxacin	NS	64	76.2	20	23.8	3.40 (1.95–5.97)	0.000	5.37 (1.29–22.38)	0.02
	S	122	48.4	130	51.6	1			
HLGR	NS	34	87.2	5	12.8	6.49 (2.47–17.04)	0.000	1.60 (0.22–11.71)	0.64
	S	152	51.2	145	48.8	1			
HLSR	NS	82	87.2	12	12.8	9.07 (4.70–14.49)	0.000	1.67 (0.33–8.43)	0.54
	S	104	43	138	57	1			
Linezolid	NS	172	97.7	4	2.3	448 (144–1392)	0.000	540 (134–2175)	0.000
	S	14	8.8	146	91.2	1			

(NS: Non-Susceptible, S: Susceptible, HLGR: High-level gentamicin resistance, HLSR: High-level streptomycin resistance).

## Data Availability

Data is contained within the article or Supplementary Materials.

## Supplementary Materials

The following supporting information can be downloaded at: https://www.mdpi.com/article/10.3390/antibiotics12060954/s1. Supplemental Table S1. Resistance patterns of *E. faecalis* isolates.
