# Supplementary material for: Prevalence and Associated Factors of optrA-Positive-Enterococcus faecalis in Different Reservoirs around Farms in Vietnam
Source: Antibiotics (Basel). 2023 May 24;12(6):954. doi: 10.3390/antibiotics12060954 (PMC10294904; doi:10.3390/antibiotics12060954)
Supplement: Supplementary file 1 [file antibiotics-12-00954-s001.zip › antibiotics-2342954-supplementary.pdf]

Supplemental Table 1. Resistance patterns of *E. faecalis* isolates

No. exhibiting antimicrobial resistance	Resistance patterns	No. of isolates			
		optrA positive (n = 186)		*optrA* negative (n=150)	
		n	%	n	%
10	E-TE-MN-DXT-C-HLGR-HLST-CIP-LEV-LNZ	2	1.1	0	0.0
	**Total**	**2**	**1.1**	**0**	0.0
9	E-TE-DXT-C-HLGR-HLSR-CIP-LEV-LNZ	1	0.5	0	0.0
	E-TE-MN-C-HLGR-HLSR-CIP-LEV-LNZ	1	0.5	0	0.0
	E-TE-MN-DXT-C-HLGR-CIP-LEV-LNZ	1	0.5	0	0.0
	E-TE-MN-DXT-C-HLGR-HLSR-CIP-LNZ	3	1.6	0	0.0
	E-TE-MN-DXT-C-HLSR-CIP-LEV-LNZ	13	7.0	0	0.0
	**Total**	**19**	**10.2**	**0**	0.0
8	E-TE-DXT-C-HLGR-HLSR-CIP-LEV	1	0.5	0	0.0
	E-TE-DXT-C-HLGR-HLSR-CIP-LNZ	1	0.5	0	0.0
	E-TE-DXT-C-HLSR-CIP-LEV-LNZ	3	1.6	0	0.0
	E-TE-MN-C-HLSR-CIP-LEV-LNZ	1	0.5	0	0.0
	E-TE-MN-DXT-C-CIP-LEV-LNZ	8	4.3	0	0.0
	E-TE-MN-DXT-C-HLGR-CIP-LNZ	0	0.0	1	0.7
	E-TE-MN-DXT-C-HLGR-HLSR-LNZ	8	4.3	0	0.0
	E-TE-MN-DXT-C-HLSR-CIP-LNZ	3	1.6	0	0.0
	**Total**	**25**	**13.4**	**1**	**0.7**
7	E-TE-C-HLGR-CIP-LEV-LNZ	1	0.5	0	0.0
	E-TE-C-HLSR-CIP-LEV-LNZ	1	0.5	0	0.0
	E-TE-DXT-C-CIP-LEV-LNZ	1	0.5	0	0.0
	E-TE-MN-DXT-C-CIP-LNZ	2	1.1	0	0.0
	E-TE-MN-DXT-C-HLGR-LNZ	4	2.2	0	0.0
	E-TE-MN-DXT-C-HLGR-HLSR	1	0.5	1	0.7
	E-TE-MN-DXT-C-HLSR-CIP	0	0	1	0.7
	E-TE-MN-DXT-C-HLSR-LNZ	21	11.3	0	0.0
	E-TE-MN-DXT-HLGR-HLSR-LNZ	3	1.6	0	0.0
	**Total**	**34**	**18.3**	**2**	**1.3**
6	E-TE-DXT-C-CIP-LEV	0	0.0	1	0.7
	E-TE-DXT-C-HLGR-LNZ	1	0.5	0	0.0
	E-TE-DXT-C-HLSR-LNZ	4	2.2	0	0.0
	E-TE-DXT-HLGR-HLSR-LNZ	1	0.5	0	0.0

	E-TE-MN-DXT-C-CIP	2	1.1	1	0.7
	E-TE-MN-DXT-C-LEV	0	0.0	1	0.7
	E-TE-MN-DXT-C-LNZ	31	16.1	2	1.3
	E-TE-MN-DXT-C-HLSR	1	0.5	5	3.3
	E-TE-MN-DXT-HLGR-HLSR	0	0.0	1	0.7
	E-TE-MN-DXT-HLSR-LNZ	2	1.1	0	0.0
	TE-MN-DXT-C-CIP-LNZ	1	0.5	0	0.0
	TE-MN-DXT-C-HLSR-LNZ	1	0.5	0	0.0
	**Total**	**44**	**23.7**	**11**	**7.3**
5	E-TE-C-CIP-LEV	1	0.5	1	0.7
	E-TE-DXT-C-LNZ	11	5.9	0	0.0
	E-TE-DXT-C-HLSR	1	0.5	0	0.0
	E-TE-HLGR-HLSR-LNZ	1	0.5	0	0.0
	E-TE-MN-C-HLGR	0	0.0	1	0.7
	E-TE-MN-DXT-C	8	4.3	9	6.0
	E-TE-MN-DXT-LNZ	3	1.6	0	0.0
	E-TE-MN-DXT-HLSR	1	0.5	1	0.7
	TE-MN-DXT-C-LEV	1	0.5	0	0.0
	TE-MN-DXT-C-LNZ	1	0.5	0	0.0
	TE-MN-DXT-HLSR-LNZ	1	0.5	0	0.0
	**Total**	**29**	**15.6**	**12**	**8.0**
4	E-TE-C-LNZ	1	0.5	0	0.0
	E-TE-DXT-C	1	0.5	6	4.0
	E-TE-DXT-HLSR	0	0	1	0.7
	E-TE-MN-DXT	1	0.5	16	10.7
	E-TE-HLSR-LNZ	1	0.5	0	0.0
	TE-DXT-C-LNZ	2	1.1	0	0.0
	TE-MN-DXT-C	0	0.0	3	2.0
	TE-MN-DXT-HLGR	0	0.0	1	0.7
	TE-MN-DXT-LNZ	7	3.8	1	0.7
	TE-MN-DXT-HLSR	0	0.0	2	1.3
	**Total**	**13**	**7.0**	**30**	**20.0**
3	C-HLGR-LNZ	1	0.5	0	0.7
	DXT-C-LNZ	1	0.5	0	0.7
	E-TE-DXT	0	0.0	1	0.0
	HLGR-HLSR-LNZ	2	1.1	0	1.3
	TE-DXT-C	0	0.0	3	0.0
	TE-DXT-LNZ	1	0.5	0	0.7

	TE-MN-DXT	1	0.5	25	0.7
	TE-MN-LNZ	1	0.5	0	0.7
	**Total**	**7**	**3.8**	**29**	**19.3**
2	E-C	0	0.0	1	0.7
	E-LNZ	0	0.0	1	0.7
	E-TE	0	0.0	3	2.0
	HLGR-LNZ	1	0.5	0	0.0
	HLSR-LNZ	2	1.1	0	0.0
	TE-DXT	1	0.5	11	7.3
	TE-LNZ	2	1.1	0	0.0
	TE-MN	0	0.0	3	2.0
	**Total**	**6**	**3.2**	**19**	**12.7**
1	CIP	0	0.0	1	0.7
	DXT	0	0.0	2	1.3
	LNZ	5	2.7	0	0.0
	TE	0	0.0	14	9.3
	**Total**	**5**	**2.7**	**17**	**11.3**
0		**2**	**1.1**	**29**	**19.3**
MDR		**160**	**86.0**	**37**	**24.7**

(*TE: tetracyclin, MN: minocyclin, DXT: doxycyclin, E: erythromycin, C: chloramphenicol, CIP: ciprofloxacin, LEV: levofloxacin, HLGR: high level gentamicin resistance, HLSR: high level streptomycin resistance, LNZ: linezolid*)
